# Vitamin K_2_ in Managing Nocturnal Leg Cramps: A Randomized Clinical Trial

**DOI:** 10.1001/jamainternmed.2024.5726

**Published:** 2024-10-28

**Authors:** Jing Tan, Rui Zhu, Ying Li, Li Wang, Shigeng Liao, Lin Cheng, LingXiu Mao, Dan Jing

**Affiliations:** 1 The Third People’s Hospital of Chengdu, Chengdu, China; 2 School of Medicine, North Sichuan Medical College, Nanchong, China; 3 Department of Neurology, Affiliated Hospital of North Sichuan Medical College, Nanchong, China

## Abstract

**Importance:**

Currently, there are no treatments for nocturnal leg cramps (NLCs) that have been proven to be both safe and effective. Seeking safe and effective approaches for managing NLCs is of crucial importance.

**Objective:**

To determine whether vitamin K_2_ is better than placebo in managing NLCs.

**Design, Setting, and Participants:**

This multicenter, double-blind, placebo-controlled randomized clinical trial was conducted in China between September 2022 and December 2023. This study used a volunteer sample comprising community-dwelling individuals 65 years and older with 2 or more documented episodes of NLCs during 2 weeks of screening. Researchers performed a history and physical screening of candidates recruited from the community through advertisements, and eligible participants were randomized in a 1:1 ratio to receive vitamin K_2_ or a placebo for 8 weeks.

**Interventions:**

Patients orally took capsules containing either vitamin K_2_ (menaquinone 7), 180 μg, or a similar-looking placebo every day for 8 weeks. The study products were custom manufactured to have identical packaging and for the capsules to have matching appearance and identical excipients that shared similar taste and weight.

**Main Outcomes and Measures:**

The primary outcome was the mean number of NLCs per week between the vitamin K_2_ and the placebo group. Secondary outcomes included the duration of muscle cramps measured in minutes and the severity of muscle cramps assessed using an analog scale ranging from 1 to 10.

**Results:**

Among the 310 participants, 111 participants were excluded. Of the 199 enrolled individuals, 108 (54.3%) were female, and the mean (SD) age was 72.3 (5.5) years. A total of 103 patients (51.8%) were randomly assigned to receive vitamin K_2_ and 96 (48.2%) were assigned to placebo. The mean (SD) baseline weekly frequency of cramps was comparable in both the vitamin K_2_ group (2.60 [0.81]) and the placebo group (2.71 [0.80]). During the 8-week intervention, the vitamin K_2_ group experienced a reduction in the mean (SD) weekly frequency of cramps to 0.96 (1.41). Meanwhile, the placebo group maintained mean (SD) weekly frequency of cramps at 3.63 (2.20). The between-group difference was statistically significant (difference, −2.67; 95% CI, −2.86 to −2.49; *P* < .001). The vitamin K_2_ group had a more significant mean (SD) reduction in NLC severity (−2.55 [2.12] points) compared with the placebo group (−1.24 [1.16] points). The vitamin K_2_ group exhibited a more pronounced mean (SD) decrease in the duration of NLCs (−0.90 [0.88] minutes) than the placebo group (−0.32 [0.78] minutes). No adverse events related to vitamin K_2_ use were identified.

**Conclusions and Relevance:**

This randomized clinical trial showed that vitamin K_2_ supplementation significantly reduced the frequency, intensity, and duration of NLCs in an older population with good safety.

**Trial Registration:**

ClinicalTrials.gov Identifier: NCT05547750

## Key Points

**Question:**

Can vitamin K_2_ significantly reduce the frequency, duration, and severity of nocturnal leg cramps (NLCs) in the older population?

**Findings:**

In this randomized clinical trial involving 199 participants 65 years and older with NLCs, those who received vitamin K_2_ experienced a significant reduction in the mean frequency of cramps per week compared with the placebo group.

**Meaning:**

This trial suggests that vitamin K_2_ might be an effective strategy for managing NLCs in older individuals with good safety profile.

## Teaser

This randomized clinical trial evaluates whether vitamin K_2_ is better than placebo in managing nocturnal leg cramps.

## Introduction

Approximately 50% to 60% of adults experience nocturnal leg cramps (NLCs) in their lifetime. Among those with NLCs, approximately 20% of individuals experiencing bothersome physical symptoms may encounter significant levels of distress and insomnia, which consequently leads them to pursue medical intervention.^1^ However, there is limited evidence supporting the use of specific medications (such as magnesium and calcium channel blockers) for managing NLCs.^1,2^ Quinine, which used to be effective in treating NLCs, is no longer recommended due to its severe adverse effects.^3^ The diversity of treatments for NLCs without robust evidence highlights the uncertainty in managing these cramps in primary care settings, emphasizing the importance of prioritizing the safety of potential therapeutic interventions.^4^ Our previous study has shown that vitamin K_2_ was effective in reducing the frequency, severity, and duration of dialysis-related muscle cramps with a good safety profile.^5^ In this current multicenter, double-blind randomized clinical trial, we aimed to investigate the efficacy and safety of vitamin K_2_ in managing NLCs.

## Methods

### Study Design

This multicenter, double-blind, placebo-controlled randomized clinical trial enrolled older individuals 65 years and older with 2 or more documented episodes of NLCs during 2 weeks of screening. Participants were randomized to receive vitamin K_2_ (menaquinone 7 [MK-7]), 180 μg, or a similar-looking placebo capsule every day for 8 weeks in a 1:1 ratio. The primary outcome was the mean number of NLCs per week in the vitamin K_2_ and placebo arms. Secondary outcomes included the duration of muscle cramps measured in minutes and the severity of muscle cramps assessed using an analog scale ranging from 1 to 10. The ethics committees of Third People’s Hospital of Chengdu and Affiliated Hospital of North Sichuan Medical College approved the study. The committees monitored the study procedure and ensured the safety of participants. All participants in the study were given clear information about the study, and all of them provided written informed consent. More details regarding this study protocol have been published previously.^6^ The trial protocol can be found in Supplement 1, and the statistical analysis plan can be found in Supplement 2. This study followed the Consolidated Standards of Reporting Trials (CONSORT) reporting guideline.

### Participants

All participants were recruited through recruitment advertisements in Third People’s Hospital of Chengdu and Affiliated Hospital of North Sichuan Medical College between September 2022 and December 2023. Potential participants experiencing NLCs willing to participate in this study were instructed to contact the research assistant, after which the researchers conducted a medical history interview to screen the participants. A history and physical examination were usually sufficient to differentiate NLCs from other conditions, such as restless legs syndrome, claudication, myositis, and peripheral neuropathy.^1,7^ Participants were invited to undergo a physical examination by the researchers (J.T. and L.W.) to confirm NLCs diagnosis and assess eligibility for participation. Inclusion criteria encompassed individuals 65 years and older with unexplained cramps that occurred twice or more in 2 weeks. Exclusion criteria referred to cramps caused by specific metabolic diseases and specific neuropathies (hypothyroidism, hemodialysis, hypoglycemia, alcoholism, amyotrophic lateral sclerosis, poliomyelitis complications, lumbar spinal stenosis, Parkinson disease, radiculopathies, and motor neuron diseases), malignant tumors, use of diuretics, vitamin K antagonist, and vitamin K_2_ within 2 months before enrollment. Informed consent was obtained from eligible participants after medical evaluation.

### Randomization and Blinding

Eligible, consenting participants were randomly assigned to vitamin K_2_ and placebo at a ratio of 1:1, using a computer-generated randomization scheme for all study centers without stratification. Participants and trial team members responsible for recruitment and monitoring were all blinded to group assignment. The study product was an over-the-counter supplement that was given to participants free of charge. The study products, custom manufactured by Sungen Biotech, featured identical packaging, capsules with matching appearance, and identical excipients that shared same taste and weight. Bottles were sequentially numbered before the commencement of the study.

### Procedures

Following the collection of demographic and clinical characteristics of the enrolled participants, they were given study products containing either vitamin K_2_ capsules or placebo capsules. Participants were instructed to take 1 capsule orally every night and to record the cramping events, duration, and level of pain experienced. Research assistants called the participants weekly to collect their self-reports, inquire about adverse reactions, remind them to continue using, and ensure record maintenance.

### Outcomes and Data Management

The primary outcome was the mean number of NLCs attacks per week; during the 8-week investigation, the differences in the frequency of attacks were recorded and compared between the vitamin K_2_ and placebo groups. Secondary outcomes included the duration of muscle cramps measured in minutes and the severity of muscle cramps assessed using an analog scale ranging from 1 to 10.

Data were collected at baseline and every week after random assignment. Phone calls from research assistants were programmed each week to collect the participants’ reports on cramping. Researchers recorded the cause and date of suspension. All data analyses were performed according to the intention-to-treat principle, and the analysis, data collection, and processing were blinded with respect to treatment group assignment. Randomized participants who did not complete the study were included in their assigned groups for the primary analysis. Mean imputations were used for missing data during the treatment phase.

### Statistical Analysis

It was calculated that a sample size of 200 participants was needed to provide at least 90% power with a significance level of 5%, assuming a mean reduction between the vitamin K_2_ group and the placebo group of 3.7 events with an SD of 8 during the intervention period. Continuous data were expressed as arithmetic means with SDs. Categorical variables were expressed as counts with frequencies. We performed a comparative analysis of baseline characteristics between the groups, using the χ^2^ test for categorical variables and *t* test for continuous variables. The study assessed the treatment effects on primary outcome variables using *t* test analysis. Significance was set at *P* < .05, and all *P* values were 2-tailed. All analyses were conducted using SPSS version 25 (IBM).

## Results

**Figure 1.**
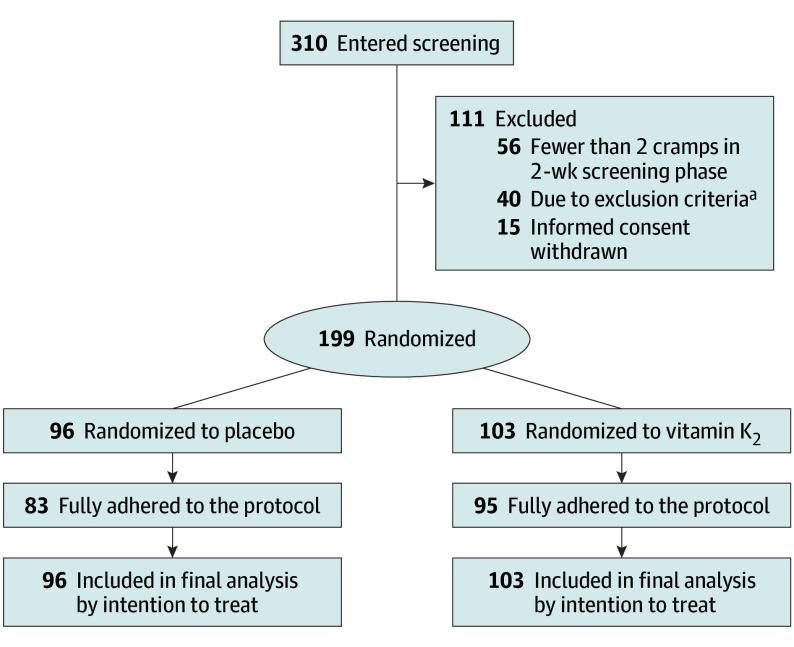
Study Flow Diagram ^a^A total of 26 candidates were excluded due to cramps caused by Parkinson disease, hypothyroidism, liver cirrhosis, or lumbar spinal stenosis; 14 candidates were excluded due to their recent (within 2 months) intake of vitamin K antagonist or vitamin K_2_ prior to enrollment.

**Figure 2.**
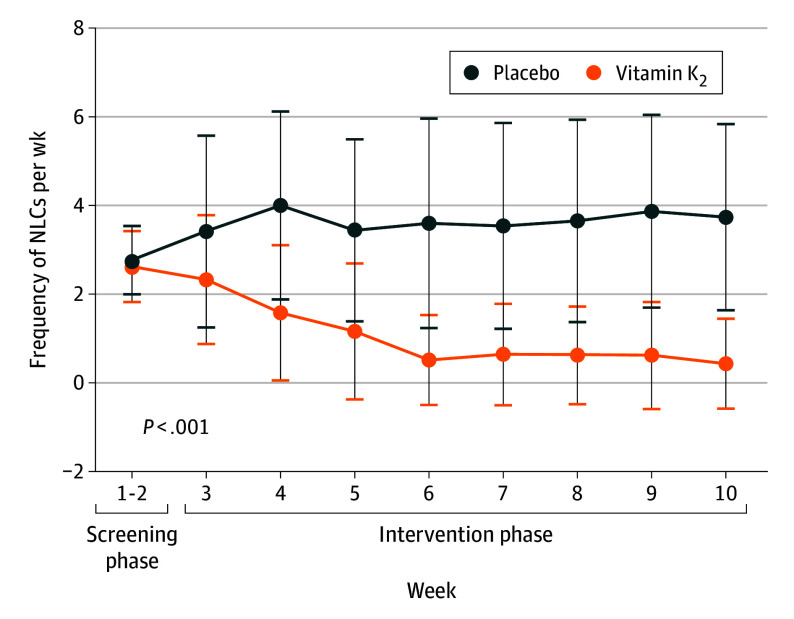
Frequency of Nocturnal Leg Cramps (NLCs) per Week Between Treatment Groups During the Study The values at the screening phase represent the mean value per week during the 2-week screening phase. Values at the intervention phase indicates real count of each time points. The *P* value provides a statistical measure of the comparison between the mean frequency per week between the groups during the intervention phase. Error bars indicate SDs.

A total of 310 candidates underwent screening at 2 centers between October 2022 and December 2023. Among the 310 participants, 111 were excluded—56 due to not meeting the inclusion criteria, 40 due to meeting exclusion criteria, and 15 due to withdrawal. Of the 199 enrolled individuals, 108 (54.3%) were female, and the mean (SD) age was 72.3 (5.5) years. A total of 103 patients (51.8%) were randomly assigned to receive vitamin K_2_ and 96 (48.2%) were assigned to placebo. Treatment compliance was 86.5% (83 of 96) in the placebo group and 92.2% (95 of 103) in the vitamin K_2_ group (Figure 1). The baseline characteristics of the participating individuals, including their concomitant chronic diseases, were similar between the treatment groups (Table 1). At baseline, the mean (SD) number of NLCs was comparable in both the vitamin K_2_ group (2.60 [0.81]) and the placebo group (2.71 [0.80]). Over the 8-week intervention period, the vitamin K_2_ group demonstrated a marked reduction in cramp frequency, reaching a mean (SD) of 0.96 (1.41). In contrast, the placebo group exhibited a persistent mean (SD) cramp frequency of 3.63 (2.20). The difference in cramp frequency at intervention phase between the vitamin K_2_ and placebo groups was statistically significant (difference, −2.67; 95% CI, −2.86 to −2.49; *P* < .001) (Figure 2; Table 2). The between-group difference became significant since the first week of the intervention (eTable 1 in Supplement 3). The mean (SD) changes in NLCs events from baseline to the intervention phase in the vitamin K_2_ group (−1.64 [1.57]) were significantly different compared with the changes in the placebo group (0.92 [2.04]; *P* < .001) (eTable 2 in Supplement 3). The duration and the pain intensity decreased during the intervention phase for the vitamin K_2_ and placebo groups. The vitamin K_2_ group showed a greater mean (SD) reduction in NLC severity (−2.55 [2.12]) compared with the placebo group (−1.24 [1.16]). Furthermore, the vitamin K_2_ group exhibited a more pronounced mean (SD) decrease in the duration of NLC (−0.90 [0.88] minutes) than the placebo group (−0.32 [0.78] minutes) (eTable 2 in Supplement 3). No adverse events related to vitamin K_2_ were identified.

**Table 1.** Baseline Characteristics of All Randomized Participants

Characteristic	Total (N = 199)	Vitamin K_2_ (n = 103)	Placebo (n = 96)
Sex, No. (%)			
Female	108 (54.3)	52 (50.5)	56 (58.3)
Male	91 (45.7)	51 (49.5)	40 (41.7)
Age, mean (SD), y	72.3 (5.5)	72.8 (5.5)	71.8 (5.5)
Height, mean (SD), cm	158.5 (6.1)	158.4 (6.1)	158.5 (6.2)
Weight, mean (SD), kg	52.5 (5.6)	52.0 (5.4)	53.1 (5.8)
Hypertension, No. (%)	136 (68.3)	74 (71.8)	62 (64.6)
Diabetes, No. (%)	101 (50.8)	55 (53.4)	46 (47.9)
Serum creatinine, mean (SD), mg/dL	0.89 (0.37)	0.87 (0.21)	0.91 (0.49)

SI conversion factor: To convert creatinine to μmol/L, multiply by 88.4.

**Table 2.** Study Outcomes

Outcome	Mean (SD)		Between-group difference, mean (95% CI)	*P* value
	Vitamin K_2_ (n = 103)	Placebo (n = 96)		
Primary outcome				
Frequency of NLCs per wk	0.96 (1.41)	3.63 (2.20)	−2.67 (−2.86 to −2.49)	<.001
Secondary outcomes				
Duration of NLCs, min	0.25 (0.47)	0.98 (0.98)	−0.73 (−0.80 to −0.65)	NA
Severity of NLCs^a^	1.12 (1.82)	2.08 (1.72)	−0.97 (−1.14 to −0.79)	NA

Abbreviations: NA, not applicable; NLCs, nocturnal leg cramps.

^a^ Determined using an analog scale ranging from 1 to 10.

## Discussion

NLC is a common nocturnal disorder that can be quite challenging to manage due to uncertainties related to precise pathophysiology. There are limited recognized intervention measures that have been proven both safe and effective.^1,2^ Given the generally benign characteristics of NLCs, treatment modality must be both effective and safe, thus minimizing the risk of iatrogenic harm. Therefore, exploring safe and effective treatments for NLCs is important. This multicenter, placebo-controlled randomized clinical trial aimed to evaluate the effectiveness and safety of vitamin K_2_ supplementation in managing NLCs. This study was based on our previous research, which revealed the efficacy of vitamin K_2_ in relieving hemodialysis-related muscle cramps.^4^ To our knowledge, this is the first study that explored the use of vitamin K_2_ specifically for treating NLCs. Our results demonstrated that daily vitamin K_2_ supplementation alleviates muscle cramps in older individuals affected by NLCs, manifested by decreased frequency, shortened duration, and weakened intensity. Notably, cramping frequency was significantly reduced compared with the placebo group, starting from the first week of intervention with vitamin K_2_.

Vitamin K_2_ is a fat-soluble vitamin involved in carboxylation that also activates several vitamin K–dependent proteins. In addition to its role in coagulation, vitamin K–dependent proteins are involved in vascular calcification and osteoporosis physiology.^8,9,10,11^ Despite extensive research on the mechanisms by which vitamin K contributes to bone and cardiovascular health, the understanding of how vitamin K affects muscle remains significantly limited. Previous clinical studies have shown that vitamin K acupuncture point injection can alleviate the pain of menstrual cramps in patients with primary dysmenorrhea.^12^ In vitro study provided a possible mechanism of the anticontraction effect of vitamin K.^13^ Vitamin K causes myometrial relaxation by inhibiting calcium intake from the external medium, an action mediated by blocking the voltage-dependent calcium channels and thus attenuating intracellular calcium levels in muscle cells.^13^

Vitamin K_2_ (MK-7) has been well-documented to be a safe supplement, as the lack of adverse effects in healthy humans precluded the World Health Organization and the Food and Agriculture Organization of the United Nations from setting a tolerable upper intake level for vitamin K_2_.^14^ No adverse events related to vitamin K_2_ were observed among our participants, thereby demonstrating the safety of vitamin K_2_ application in the older population with NLCs. It is crucial to note that vitamin K_2_ has the potential to compromise the anticoagulant effectiveness of warfarin, a widely prescribed medication for older individuals. Therefore, vitamin K_2_ is not recommended for those undergoing warfarin therapy.

### Limitation

This study has limitations. Nocturnal muscle cramping, also known as sleep-related leg cramping, impairs sleep quality due to the discomfort associated with the cramps. The potential efficacy of vitamin K_2_ intervention in mitigating cramping and subsequently enhancing sleep quality has garnered significant research interest. However, this study did not investigate the quality of life or sleep. Considering that the inclusion of daily life and sleep quality questionnaires for older adults might compromise their adherence to the study protocol, quality of life and sleep outcomes were not addressed in this trial protocol. Future studies are warranted to clarify the impact of vitamin K_2_ on quality of life and sleep in patients with NLCs.

Another limitation of this study is the relatively mild nature of NLCs experienced by our participants, who were all community-dwelling individuals. The mean frequency of NLCs was 2.6 to 2.7 per week, with a low mean severity rating of 3.3 to 3.6 on a scale ranging from 1 to 10 and a mean duration of 1.15 to 1.3 minutes. Although our previous study found that vitamin K_2_ effectively reduced the more severe cramping symptoms related with hemodialysis,^5^ further research is required to confirm whether vitamin K_2_ can demonstrate efficacy in other populations with more pronounced symptoms.

## Conclusions

This randomized clinical trial demonstrated that vitamin K_2_ supplementation significantly reduced the frequency, intensity, and duration of NLCs in an older population with good safety. Due to the safety profile of vitamin K_2_, clinical trials are encouraged to confirm the efficacy of cramping management and its impact on the quality of life and sleep in patients with NLCs.

## Notes

Supplement 1. Trial Protocol

Supplement 2. Statistical Analysis Plan

Supplement 3. **eTable 1.** Overview of Outcomes Between the Vitamin K_2_ and the Placebo at Different Time Points **eTable 2.** Magnitude of Change From Baseline to Intervention Between Vitamin K_2_ and Placebo Group

Supplement 4. Data Sharing Statement
