# Supplementary material for: Vitamin K2 in Managing Nocturnal Leg Cramps: A Randomized Clinical Trial
Source: JAMA Intern Med. 2024 Oct 28;184(12):1443–7. doi: 10.1001/jamainternmed.2024.5726 (PMC11581596; doi:10.1001/jamainternmed.2024.5726)
Supplement: Supplement 1. — Trial Protocol [file jamainternmed-e245726-s001.pdf]

Effect of vitamin K2 in the treatment of nocturnal leg cramps in the older population: Study protocol of a randomized, double-blind, controlled trial

Protocol No.: [2022]S-17

Version:2.0

Date of Protocol Version: July/01/2022

Principal Investigator: Dr. Jing Tan

The Third People's Hospital of Chengdu Ethics Committees Review Approval Number: [2022]S-17

Affiliated Hospital of North Sichuan Medical College Ethics Committees Review Approval Number:2022ER386-1

Approvals

Principal Investigator Agreement:

I have carefully read and understood the provisions of this protocol and I am prepared to follow them in every detail in the conduct of this study.

Principal Investigator Signature: ____________ Date: 2022.7.1

Protocol Modifications History:

Date	Protocol No (Ver)	Protocol Changes
June/15/2022	[2022]S-17 V 2.0	1. Modifications for "Handling of dropouts" as requested by the Ethics Committees

SYNOPSIS:

STUDY TITLE: Effect of vitamin K2 in the treatment of nocturnal leg cramps in the older population: Study protocol of a randomized, double-blind, controlled trial.

SPONSOR: The Third People's Hospital of Chengdu

INVESTIGATIONAL PRODUCT AND FORMULATION: Vitamin K2 (Ingredients: Vitamin K2 obtained through fermentation, providing 180μg of vitamin K2, excipients: microcrystalline cellulose, silicon dioxide, magnesium stearate), Sungen Biotech Co., Ltd., Shantou, China.

STUDY PHASE: 4

INDICATION: Nocturnal Leg Cramps (NLC).

OBJECTIVES: The objective of the present study is to evaluate the efficacy and safety of Vitamin K2 in the older population with NLC.

TRIAL DESIGN: A multicenter center, prospective, randomized, double blind, placebo controlled clinical trial

SAMPLE SIZE AND TREATMENT GROUPS: A total of 200 subjects will randomly be assigned to two study arms employing a 1:1 randomization scheme. Each randomized individual will be given once daily either Vitamin K2 or a similarly looking placebo for a treatment period of 8 weeks.

CLINICAL TRIAL DURATION: Two weeks of eligibility screening followed by 8-week double blinded treatment.

INCLUSION CRITERIA:

1. Unexplained cramps occurred two or more times in 2weeks.
2. Age≥65-year-old.

EXCLUSION CRITERIA:

1. Cramps caused by specific metabolic diseases and specific neuropathies (hypothyroidism, hemodialysis, hypoglycemia, alcoholism, amyotrophic lateral sclerosis, poliomyelitis complications, lumbar spinal stenosis, Parkinson’s disease, radiculopathies, and motor neuron diseases).
2. Suffering from malignant tumors (breast cancer, prostate cancer, lymphoma, and multiple

myeloma).

3. Taking diuretics, or vitamin K antagonist.

4. Taking supplements with vitamin K2 within 2months before enrollment.

DOSAGE OF INVESTIGATIONAL PRODUCT: Vitamin K2 (Ingredients: Vitamin K2 obtained through fermentation, providing 180μg of vitamin K2, excipients: microcrystalline cellulose, silicon dioxide, magnesium stearate), Sungen Biotech Co., Ltd., Shantou, China.

CONTROL PRODUCTS: Similarly looking placebo with identical packaging and capsules that had matching appearance, taste, and weight.

MODALITIES OF DRUG ADMINISTRATION: Oral administration once daily for 8 weeks.

MAIN STUDY PARAMETERS:

Number of episodes of NLC; measured as evens every week

Severity of NLC; measured in minutes

Duration of NLC; measured using a 1-10 analog scale.

CLINICAL TRIAL PROCEDURES:

Participants were instructed to take one capsule orally every night and to document the cramping events, duration, and level of pain experienced.

The number, duration and severity of NLC will be recorded in the trial compared with Vitamin K2 and placebo treated individuals during 8 weeks of treatment.

Study assistants called the participants weekly to collect their self-reports, inquire about adverse reactions, remind them to continue using, and ensure record maintenance.

STUDY METHODS:

The study was designed as a multi-center, prospective, randomized, double blind, placebo-controlled, 2-arms parallel-groups, clinical trial, designed and powered to assess the efficacy of oral administration of Vitamin K2 in subjects suffering from NLC in older population.

PRIMARY ENDPOINT:

The mean number of NLCs attacks per week (During the 8-week investigation, the differences in the frequency of attacks will be recorded and compared between vitamin K2

and placebo arms).

KEY SECONDARY ENDPOINTS:

1.The severity of muscle cramps using a 1–10 analog scale (During the 8-week investigation, pain severity during attacks will be recorded and compared between vitamin K2 and placebo arms). The participants will be asked to record the severity of cramping with a 1–10 analog scale. That is, 1–3 points, mild pain, tolerable, does not affect sleep; 4–6 points, moderate pain, affects sleep, also tolerable; 7–10 points, sharp pain, intolerable.

SAMPLE SIZE RATIONALE:

It was calculated that a sample size of 200 participants was needed to provide at least 90% power with a significance level of 5%, assuming a mean reduction between the vitamin K2 group and the placebo group of 3.7 events with a standard deviation of 8 during the intervention period.

STATISTICAL METHODS:

SPSS statistical software is used for data analysis. Non-parametric tests (Mann–Whitney U test) are used for non-normal data. Results are given as medians with 95% confidence intervals (CI). Approximately normally distributed data are described as means and standard deviations. Other statistical tests used include Student’s t-tests for continuous normally distributed data and Chi-Square tests for categorical data are stated with the results.

STUDY DESIGN AND FLOW CHART.

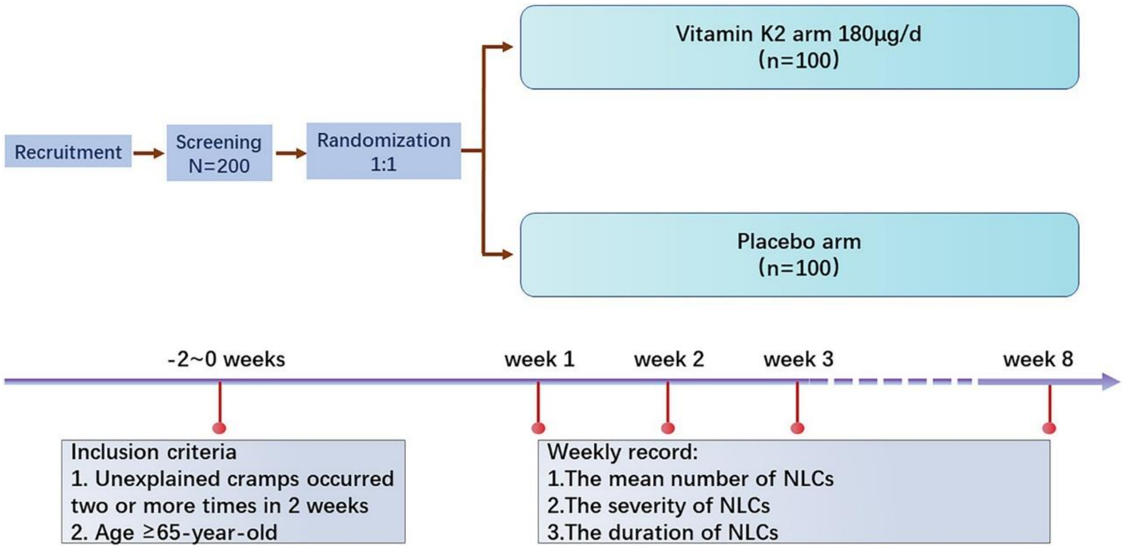

STUDY EVALUATION PROCEDURES AND TIMELINE.

Trail phase	Screening −2week	Baseline 0week	Intervention							
			1week	2week	3week	4week	5week	6week	7week	8week
Sign the informed consent form	×									
Determine eligibility	×									
Obtain medical and demographic data		×								
Fill in the general information	×									
Comorbidities and treatment			×	×	×	×	×	×	×	×
Outcome measures										
The mean number of NLCs			×	×	×	×	×	×	×	×
The severity of NLCs			×	×	×	×	×	×	×	×
The duration of NLCs			×	×	×	×	×	×	×	×
Physical examination						×				×

Introduction:

Nocturnal leg cramps (NLCs) are spontaneous contractions of muscles. The gastrocnemius is commonly involved (1), lasting from a few seconds to a few minutes (2). Patients might wake up with pain during attacks, making it difficult to sleep for a short period. It commonly occurs >60-year-old (3). The medical history and physical examination are usually sufficient to differentiate nocturnal leg cramps from other conditions, such as restless legs syndrome, claudication, myositis, and peripheral neuropathy. Factors that may lead to leg cramps attacks include hemodialysis, electrolyte imbalance, metabolic disorders, and congenital disorders (4). The cramps can be relieved by passive stretching of the gastrocnemius and deep tissue massage, but such prevention is limited, especially in patients with refractory muscle cramps (5). Quinine has been shown to be effective in treating NLCs but is not

recommended by the US Food and Drug Administration due to severe side effects (6).Magnesium supplements are often used as a preventative treatment for NLCs (7, 8); however, their effectiveness is controversial (2, 9, 10). Magnesium supplements are widely marketed for the prophylaxis of NLCs since a double-blind, placebo-controlled study proved their effectiveness in pregnant women (11). However, magnesium administration did not show significant benefits in NLCs in double-blind, placebo-controlled studies (12, 13). Metaanalysis of some randomized control trials (RCTs) showed that magnesium therapy did not appear to be effective in the treatment of NLCs in the general population, but may have a negligible effect in pregnant women (14). Therefore, seeking new approaches to manage NLCs is imperative.

Vitamin K is a fat-soluble vitamin involved in carboxylation and activating several dependent proteins. It is found in two isoforms (phylloquinone (vitamin K1) and menaquinone (vitamin K2)) that differ in length and degree of saturation of the side chain.In addition to their role in coagulation, vitamin K-dependent proteins are involved in vascular calcification and osteoporosis physiology. Accumulating evidence has shown the beneficial effects of vitamin K2 supplementation on cardiovascular and bone health (15).

Another study revealed that vitamin K3 relieved muscle cramps by effectuating the voltage-dependent calcium channels to release the calcium stored in the cells, thus reducing the frequency of muscular contractions (16). To the best of our knowledge, no study has yet investigated the efficacy of vitamin K in NLCs. In addition, vitamin K2 has a good safety profile compared to other medications. Our pilot study demonstrated that vitamin K2 supplementation decreases the frequency, duration, and severity of muscle cramps in hemodialysis patients (17). To further investigate the efficacy and safety of vitamin K2 in NLCs, we designed this prospective, multicenter, randomized, double blinded trial.

Hypothesis

Vitamin K2 may reduce the number, duration and severity of NLC; an improvement in the quality of life may ensue.

Objective:

To assess the effect of treatment with Vitamin K2, as compared with placebo, on frequency, duration and severity of NLC of NLC.

Methods

Study design:

A prospective, randomized, double blind, placebo controlled clinical trial.

Participants:

Two hundred individuals afflicted by NLC will be recruited for the study.

Inclusion criteria:

1. Unexplained cramps occurred two or more times in 2weeks.
2. Age≥65-year-old.

Exclusion criteria:

1. Cramps caused by specific metabolic diseases and specific neuropathies (hypothyroidism, hemodialysis, hypoglycemia, alcoholism, amyotrophic lateral sclerosis, poliomyelitis complications, lumbar spinal stenosis, Parkinson’s disease, radiculopathies, and motor neuron diseases).
2. Suffering from malignant tumors (breast cancer, prostate cancer, lymphoma, and multiple myeloma).
3. Taking diuretics, or vitamin K antagonist.
4. Taking supplements with vitamin K2 within 2months before enrollment.

Setting:

Chengdu Third People’s Hospital, Chengdu, Sichuan, China

Affiliated Hospital of North Sichuan Medical College, Nanchong, Sichuan, China

Intervention:

Each randomized individual will be oral once daily either Vitamin K2 (Ingredients: Vitamin K2

obtained through fermentation, providing 180ug of vitamin K2; Excipients in the capsule include: microcrystalline cellulose, silicon dioxide, and magnesium stearate) or a similarly looking placebo(Excipients in the capsule include: microcrystalline cellulose, silicon dioxide, and magnesium stearate), for a treatment period of 8 weeks.

Outcomes:

The primary outcome is the number of NLC during the 8 weeks of treatment period.

The secondary outcomes are: Severity of NLC; Duration of NLC.

Outcome and compliance ascertainment

During the treatment phase of the study (8 weeks), participants will be instructed to take one capsule orally every night and to document the cramping events, duration, and level of pain experienced. Study assistants will call the participants weekly to collect their self-reports, inquire about adverse reactions, remind them to continue using, and ensure record maintenance. The degree of pain will be recorded in the dairy on a scale of 0 to 10 (0=no pain, 10= intolerable pain).

Ethical Issues

The study product was an over-the-counter supplement that was given to participants free of charge. The ethics committees of Third People's Hospital of Chengdu and Affiliated Hospital of North Sichuan Medical College approved the study. The committees monitored the study procedure and ensured the safety of participants. All participants in the study were given clear information about the study, and all of them provided written informed consent.

Enrolment and Eligibility Screening

We will invite individuals who meet the criteria of being aged ≥65 and who have reported two or more NLCs within the past 2 weeks among community residents to sign up. A comprehensive description of the study rational and process will be provided and, after questioning and elaboration as requested by the candidate, a signed informed consent will be obtained.

Randomization

Eligible, consenting participants were randomly assigned to Vitamin K2 and placebo at a ratio of 1:1, using a computer-generated randomization scheme for all study centers without stratification.

Masking

Identical capsules, containing Vitamin K2 or placebo according to the randomization schedule, are prepacked in identical bottles and consecutively numbered for each enrolled individual. Both patients and investigators are blinded to treatment allocation for each individual. Participants and trial team members responsible for recruitment and monitoring were all blinded to group assignment. The study products, custom-manufactured by Sungen Biotech (Sungen Biotech Co., Ltd., Shantou, China), featured identical packaging with capsules that were matched for appearance, taste, and weight.

Definition of Participants' Validity Determination

Exclusion criteria:

Enrolled cases that meet one of the following criteria should be excluded: (1)Cases in which subjects were found not to meet the inclusion criteria after enrollment;(2) cases in which prohibited drugs and treatment methods specified in the protocol were used, treatment requirements of the trial protocol were not followed, and compliance was poor;(3) cases in which no drugs were used after enrollment;(4) cases in which no evaluable records were available after treatment.

Withdrawal criteria:

Withdrawal decided by the investigator: After taking the drug, the symptoms of the patient were aggravated. In order to protect the subject, the subject was withdrawn from the trial and received other effective treatments; during the trial, the subject experienced some comorbidities, complications or special physiological changes, which made it inappropriate

to continue the trial; during the trial, prohibited drugs or treatment methods specified in the protocol were used.

Subject withdraws from the trial voluntarily: According to the provisions of informed consent form, the subject has the right to withdraw from the trial halfway, or although the subject does not explicitly withdraw from the trial, but no longer receives treatment and testing and is lost to follow-up, it is also considered as "withdrawal"(or "drop-out"). The reasons for withdrawal should be understood as much as possible and recorded, such as: self-perceived poor efficacy; intolerable to some adverse reactions; unable to continue the clinical study due to some reasons; economic factors; or lost to follow-up without explaining the reasons, etc.

Regardless of the reason, the case record form should be retained for the case withdrawn from the trial, and the final test result should be transferred to the final result, and the full data set analysis of efficacy and adverse reactions should be performed.

Drop-out and treatment of cases

Definition of drop-out: All subjects who have completed the informed consent form and are screened and qualified to enter the randomized trial, regardless of when and why they withdraw, as long as they do not complete the observation period specified in the protocol, are drop-out cases. During the trial, if the treatment time is less than 1 course, but the symptoms have completely disappeared and the subjects stop treatment voluntarily, they are not considered as drop-out cases.

Handling of dropouts: (1) When a subject withdraws, the investigator should contact the subject as much as possible by visiting, making an appointment for follow-up, telephone, letter, etc., ask the reason, record the last treatment time, and complete the evaluation items that can be completed;(2) If the subject withdraws from the trial due to allergic reactions, adverse reactions, and ineffective treatment, the investigator should take corresponding treatment measures according to the actual situation of the subject;(3) For drop-out cases, the investigator must fill in the detailed reasons for drop-out in the case report form. Statistical analysis should be combined with the actual situation, such as adverse reactions

should be included in the statistics of adverse reactions;
The dropout rate should be controlled below 10% as far as possible.

Adverse events

Definition

Any unfavorable medical event occurring between the time the patient signed the informed consent form and was enrolled in the trial and the last follow-up visit, regardless of its causal relationship to the trial drug, was considered an adverse event.

During the trial, adverse events were recorded truthfully, including the time of occurrence, severity and duration of adverse events.

Criteria for severity of adverse events

Refer to the grading evaluation criteria for adverse drug reactions in NCI CTC Version 4. If adverse reactions not listed in the table occur, refer to the following expressions: Mild: Does not affect the normal function of the subject. Moderate: To some extent affects the normal function of the subject. Severe: Significantly affects the normal functioning of the subject.

Criteria for determining the relationship between adverse events and the trial

The investigator should assess the possible association between the adverse event and the trial drug by reference to the following criteria:

Related: the reaction occurred in chronological order of administration, the reaction was consistent with the known reaction type of the investigational product, improved after discontinuation, and reappeared after repeated administration.

Possible: the timing of the reaction corresponds to the chronology of administration, the reaction corresponds to known reaction types of the investigational drug, the patient's clinical status or other treatment modalities may also cause the reaction.

Possibly unrelated: The timing of the reaction does not correspond to the chronological order of administration, the reaction does not fit well with the known reaction type of the investigational drug, and the patient's clinical state or other treatment methods may also cause the reaction.

Irrelevant: The timing of the reaction does not correspond to the chronological order of administration, the reaction is consistent with the known reaction type of non-investigational drugs, the patient's clinical state or other treatment methods may also cause the reaction, the disease state improves or other treatment methods are discontinued, and the reaction disappears after repeated use of other treatment methods.

Unable to determine: the timing of the reaction is not clearly related to the chronological order of administration, the reaction is similar to the known reaction type of the investigational drug, and other drugs administered at the same time may also cause the same reaction.

Serious adverse event

Judgment of serious adverse event: death; life-threatening; leading to prolonged hospitalization; permanent or serious disability; leading to congenital malformation or defect.

Reporting system

Any serious adverse reactions occurring during the clinical trial or within 30 days of the last treatment, whether related to the drug or not, should be reported immediately to the project leader (PI) of the leading clinical site and the Ethics Committee by telephone within 24 hours, and the sponsor should report to the National Drug Administration.

Statistical methods:

It was calculated that a sample size of 200 participants was needed to provide at least 90% power with a significance level of 5%, assuming a mean reduction between the vitamin K2 group and the placebo group of 3.7 events with a standard deviation of 8 during the intervention period. We will perform all data analyses according to the intention to treat principle, and the analysis, data collection, and processing will be blinded with respect to treatment group assignment. Randomized participants who do not complete the study will be included in their assigned study groups for the primary analysis. Continuous data were expressed as arithmetic means ±standard deviation. Categorical variables were expressed as frequencies. We performed a comparative analysis of baseline characteristics between

the groups, utilizing the χ^2 test for categorical variables and t test for continuous variables. The study assessed the treatment effects on primary and secondary outcome variables using a repeated-measures analysis, which compared individual differences across treatment groups at different treatment phases.

Role of Investigators and Manufacturer

Principal Investigator: Dr Jing Tan, M.D.& Ph.D., Department of Haematology, Chengdu Third People's Hospital, Chengdu, Sichuan, China

Acquisition, analysis, or interpretation of data: Rui Zhu, Ying Li, Shigeng Liao, Ling cheng, Dan Jing, Lingxiu Mao; Statistical analysis: Rui zhu.

The study was conceived by Jing Tan and Li Wang. The protocol was drafted by Jing Tan and Li Wang; the final version was approved by all investigators, without any involvement of the manufacturer.

Funding:

This work was supported by The Third People’s Hospital of Chengdu Scientific Research Project (2023PI05; China Health Promotion Foundation grant(Z093001).

REFERNCES

1. Maisonneuve, H, Chambe, J, Delacour, C, Muller, J, Rougerie, F, Haller, DM, et al Prevalence of cramps in patients over the age of 60 in primary care: a cross sectional study. BMC Fam Pract. (2016) 17:111. doi: 10.1186/s12875-016-0509-9

2. Monderer, RS, Wu, WP, and Thorpy, MJ. Nocturnal leg cramps. Curr Neurol Neurosci Rep. (2010) 10:53–9. doi: 10.1007/s11910-009-0079-5

3. Lorenzo, M, Schaeffer, M, Haller, DM, and Maisonneuve, H. Treatment of nocturnal leg cramps by primary care patients over the age of 60. Fam Pract. (2018) 35:29–33. doi: 10.1093/fampra/cmx062

4. Allen, RE, and Kirby, KA. Nocturnal leg cramps. Am Fam Physician. (2012) 86:350–5.

5. Daniell, HW. Simple cure for nocturnal leg cramps. N Engl J Med. (1979) 301:216.

6. Guay, DR. Are there alternatives to the use of quinine to treat nocturnal leg cramps? Consult Pharm. (2008) 23:141–56. doi: 10.4140/tcp.n.2008.141

7. Barna, O, Lohoida, P, Holovchenko, Y, Bazylevych, A, Velychko, V, Hovbakh, I, et al A randomized, double-blind, placebo-controlled, multicenter study assessing the efficacy of magnesium oxide monohydrate in the treatment of nocturnal leg cramps.Nutr J. (2021) 20:12. doi: 10.1186/s12937-021-00747-9

8. Garrison, SR, Korownyk, CS, Kolber, MR, Allan, GM, Musini, VM, Sekhon, RK, et al Magnesium for skeletal muscle cramps. Cochrane Database Syst Rev. (2020) 9, 9:CD009402:CD009402. doi: 10.1002/14651858.CD009402.pub3 9. Young, G. Leg cramps. BMJ Clin Evid. (2015) 2009:1113. (Accessed March 26, 2009)

10. Young, GL, and Jewell, D. Interventions for leg cramps in pregnancy. Cochrane Database Syst Rev. (2002) 1:CD000121. doi: 10.1002/14651858.CD000121

11. Dahle, LO, Berg, G, Hammar, M, Hurtig, M, and Larsson, L. The effect of Oral magnesium substitution on pregnancy-induced leg cramps. Am J Obstet Gynecol. (1995) 173:175–80. doi: 10.1016/0002-9378(95)90186-8

12. Roguin Maor, N, Alperin, M, Shturman, E, Khairaldeen, H, Friedman, M, Karkabi, K, et al Effect of magnesium oxide supplementation on nocturnal leg cramps: a randomized clinical

trial. JAM
15. Villa, JKD, Diaz, MAN, Pizziolo, VR, and Martino, HSD. Effect of vitamin K in bone metabolism and vascular calcification: a review of mechanisms of action and evidences. Crit Rev Food Sci Nutr. (2017) 57:3959–70. doi: 10.1080/10408398.2016.1211616
16. Zhang, XX, Lu, LM, and Wang, L. Vitamin K3 inhibits mouse uterine contraction in vitro via interference with the calcium transfer and the potassium channels. Biochem Biophys Res Commun. (2016) 476:393–9. doi: 10.1016/j.bbrc.2016.05.132
17. Xu, D, Yang, A, Ren, R, Shan, Z, Li, YM, and Tan, J. Vitamin K2 as a potential therapeutic candidate for the prevention of muscle cramps in hemodialysis patients: a prospective multicenter, randomized, controlled, crossover pilot trial. Nutrition. (2022) 97:111608:111608. doi: 10.1016/j.nut.2022.111608
