# Supplementary material for: Vitamin K2 in Managing Nocturnal Leg Cramps: A Randomized Clinical Trial
Source: JAMA Intern Med. 2024 Oct 28;184(12):1443–7. doi: 10.1001/jamainternmed.2024.5726 (PMC11581596; doi:10.1001/jamainternmed.2024.5726)
Supplement: Supplement 2. — Statistical Analysis Plan [file jamainternmed-e245726-s002.pdf]

Statistical Analysis Plan:

Effect of vitamin K2 in the treatment of nocturnal leg cramps (VK2-NLC) in the older population: Study protocol of a randomized, double-blind, controlled trial

Version: 1.0

Date: [Feb/11/2022]

Principal Investigator: Jing Tan, MD

INTRODUCTION

The purpose of this Statistical Analysis Plan (SAP) is to provide a more detailed description of statistical methods and presentation of the study data to be used for the analysis of data generated from the clinical trial described in protocol: Effect of vitamin K2 in the treatment of nocturnal leg cramps (VK2-NLC) in the older population: Study protocol of a randomized, double-blind, controlled trail. Version 1.0 (Feb/11/2022)

This SAP was prepared by Dr.Jing Tan and Dr. Wang Li. It includes details of data handling procedures and statistical methodology. The final statistical analyses will proceed in accordance with this SAP. Any deviation from this SAP will be documented in the final clinical study report.

STUDY DESIGN

This prospective, multicenter RCT will recruit participants from two tertiary hospitals, Chengdu Third People's Hospital and Affiliated Hospital of North Sichuan Medical College, with the diagnosis of NLCs. This manuscript is according to the Standard Protocol Items: Recommendations for Interventional Trials (SPIRIT) guidelines (1). The study will conduct as a randomized controlled, double-blinded trial. The subjects screened with NLCs will be randomly assigned to two arms: the vitamin K2 arm (vitamin K2 180 μg/day) and the placebo arm. The arms would consist of an equal number of participants, and the study would be conducted double-blind between the participants and the researchers during observation. The overall framework is to compare the treatment outcomes changes between the two groups.

Participants

All participants are planned to be recruited through recruitment advertisement from September 2022 to September 2023. Potential participants suffering from NLCs are willing to participate in this study. In that case, they may contact the research assistant, who will make a medical history interview to screen the participants. Eligible

participants will be invited to participate in a physical examination to confirm NLC diagnosis and assess eligibility for participation in the study. A history and physical examination were usually sufficient to differentiate NLCs from other conditions, such as restless legs syndrome, claudication, myositis, and peripheral neuropathy(2). Participants will be invited to undergo a physical examination by the researcher to confirm NLC diagnosis and assess eligibility for participation. A research assistant will meet with the eligible participants after the medical evaluation and obtain their written informed consent. All participants' demographic variables, such as age, sex, medical history, and lifestyle (smoking and alcohol use), will be collected before intervention (baseline). Participants will also be asked relevant questions about the duration of symptoms and previous treatments.

Procedures

A total of 200 participants will be randomly assigned to the two study groups using a 1:1 randomization protocol (n = 100/group). The vitamin K2 arm takes vitamin K2 180 μg/day at bedtime and the placebo arm takes a placebo at bedtime for 8 weeks. The frequency of muscle cramps and the duration and severity of each attack in both arms will be recorded every week (Figure 1). Study assistants will call the participants weekly to collect their self-reports, inquire about adverse reactions, remind them to continue using, and ensure record maintenance.

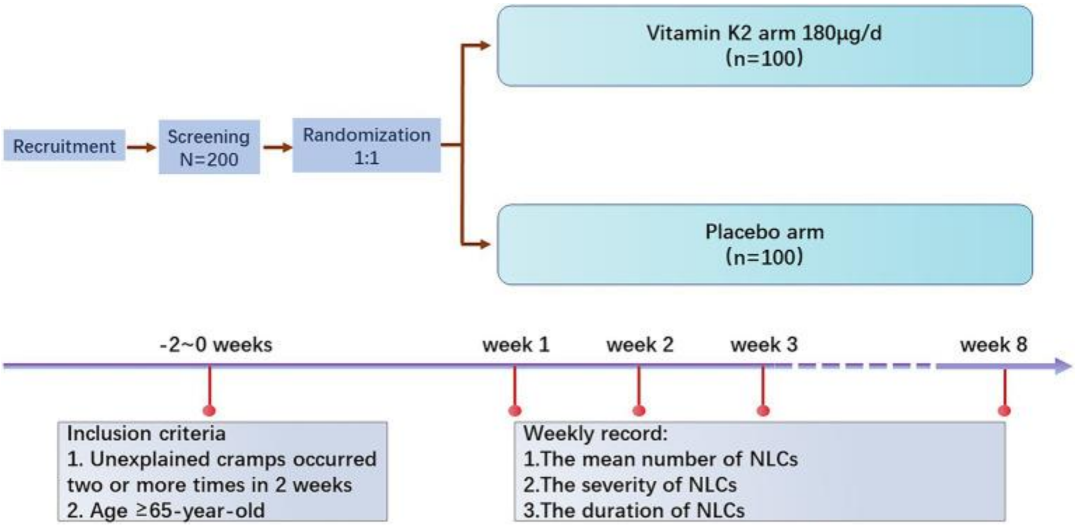

Figure1. Study design and flow chart.

Inclusion criteria: 1. Unexplained cramps occurred two or more times in 2 weeks; 2. Age ≥ 65-year-old.

Exclusion criteria: 1. Cramps caused by specific metabolic diseases and specific neuropathies (hypothyroidism, hemodialysis, hypoglycemia, alcoholism, amyotrophic lateral sclerosis, poliomyelitis complications, lumbar spinal stenosis, Parkinson's disease, radiculopathies, and motor neuron diseases). 2.Suffering from malignant tumors (breast cancer, prostate cancer, lymphoma, and multiple myeloma). 3.Taking diuretics, or vitamin K antagonist. Taking supplements with vitamin K2 within 2 months before enrollment.

Withdrawal criteria:

(1) Withdrawal decided by the investigator: After taking the drug, the symptoms of the patient were aggravated. In order to protect the subject, the subject was withdrawn from the trial and received other effective treatments; during the trial, the subject experienced some comorbidities, complications or special physiological changes, which made it inappropriate to continue the trial; during the trial, prohibited drugs or treatment methods specified in the protocol were used.

(2) Subject withdraws from the trial voluntarily: According to the provisions of informed consent form, the subject has the right to withdraw from the trial halfway, or although the subject does not explicitly withdraw from the trial, but no longer receives treatment and testing and is lost to follow-up, it is also considered as "withdrawal"(or "drop-out"). The reasons for withdrawal should be understood as much as possible and recorded, such as: self-perceived poor efficacy; intolerable to some adverse reactions; unable to continue the clinical study due to some reasons; economic factors; or lost to follow-up without explaining the reasons, etc.

(3) Regardless of the reason, the case record form should be retained for the case withdrawn from the trial, and the final test result should be transferred to the result, and the full data set analysis of efficacy and adverse reactions should be performed.

Drop-out and treatment of cases

3.1 Definition of drop-out: All subjects who have completed the informed consent form and are screened and qualified to enter the randomized trial, regardless of when

and why they withdraw, as long as they do not complete the observation period specified in the protocol, are drop-out cases. During the trial, if the treatment time is less than 2 week, but the symptoms have completely disappeared and the subjects stop treatment voluntarily, they are not considered as drop-out cases.

3.2 Handling of dropouts: (1) When a subject withdraws, the investigator should contact the subject as much as possible by visiting, making an appointment for follow-up, telephone, letter, etc., ask the reason, record the last treatment time, and complete the evaluation items that can be completed;(2) If the subject withdraws from the trial due to allergic reactions, adverse reactions, and ineffective treatment, the investigator should take corresponding treatment measures according to the actual situation of the subject;(3) For drop-out cases, the investigator must fill in the detailed reasons for drop-out in the case report form. Statistical analysis should be combined with the actual situation, such as adverse reactions should be included in the statistics of adverse reactions.

The dropout rate should be controlled below 10% as far as possible.

Data management

Data will be collected at baseline and every week after random assignment (Table 1). Phone calls from research assistants will be programmed each week to maximize participant compliance in subsequent assessments. A registered participant will be excluded from the study if exclusion criteria are detected after registration. Researchers will record the cause and date of suspension. The consent to use data collected before the participant’s withdrawal will be included in the informed consent form. We will perform all data analyses according to the intention to treat principle, and the analysis, data collection, and processing will be blinded with respect to treatment group assignment. Randomized participants who do not complete the study will be included in their assigned study groups for the primary analysis.

Trail phase	Screening −2 week	Baseline 0 week	Intervention							
			1 week	2 week	3 week	4 week	5 week	6 week	7 week	8 week
Sign the informed consent form	×									
Determine eligibility	×									
Obtain medical and demographic data		×								
Fill in the general information	×									
Comorbidities and treatment			×	×	×	×	×	×	×	×
Outcome measures										
The mean number of NLCs			×	×	×	×	×	×	×	×
The severity of NLCs			×	×	×	×	×	×	×	×
The duration of NLCs			×	×	×	×	×	×	×	×
Physical examination						×				×

Table 1. Study evaluation procedures and timeline

Sample size

It will be calculated that a sample size of 200 participants was needed to provide at least 90% power with a significance level of 5%, assuming a mean reduction between the vitamin K2 group and the placebo group of 3.7 events with a standard deviation of 8 during the intervention period.

Intervention description

Vitamin K2

Vitamin K2 is a fat-soluble vitamin involved in carboxylation that also activates several vitamin K-dependent proteins. In addition to its role in coagulation, vitamin K-dependent proteins are involved in vascular calcification and osteoporosis physiology. Despite extensive research on the mechanisms by which vitamin K contributes to bone and cardiovascular health, the understanding of how vitamin K affects muscle remains significantly limited.

Placebo

Placebo tablets appear like those of vitamin K2, which will be custom-manufactured by manufacture, featured identical packaging with capsules that matched for appearance, taste, and weight.

STUDY OBJECTIVES

To determine whether vitamin K2 is better than placebo in managing NLCs

The primary outcome: The mean number of NLCs attacks per week (During the 8-week investigation, the differences in the frequency of attacks will be recorded and

compared between vitamin K2 and placebo arms)

Secondary outcomes: Duration of muscle cramps in minutes (During the 8-week investigation, the differences in the duration of attacks will be recorded and compared between vitamin K2 and placebo arms). The severity of muscle cramps using a 1–10 analog scale (During the 8-week investigation, pain severity during attacks will be recorded and compared between vitamin K2 and placebo arms). The participants will be asked to record the severity of cramping with a 1–10 analog scale. That is, 1–3 points, mild pain, tolerable, does not affect sleep; 4–6 points, moderate pain, affects sleep, also tolerable; 7–10 points, sharp pain, intolerable.

Adverse events

Definition

Any untoward medical event occurring between the time the patient signed the informed consent form and was enrolled in the trial and the last follow-up visit, regardless of its causal relationship to the trial drug, was considered an adverse event. During the trial, adverse events were recorded truthfully, including the time of occurrence, severity and duration of adverse events.

Criteria for severity of adverse events

Refer to the grading evaluation criteria for adverse drug reactions in NCI CTC Version 4. If adverse reactions not listed in the table occur, refer to the following expressions: Mild: Does not affect the normal function of the subject. Moderate: To some extent affects the normal function of the subject. Severe: Significantly affects the normal functioning of the subject.

Criteria for determining the relationship between adverse events and the trial

The investigator should assess the possible association between the adverse event and the trial drug by reference to the following criteria:

Definitely related: the reaction occurred in chronological order of administration, the reaction was consistent with the known reaction type of the investigational product, improved after discontinuation, and reappeared after repeated administration.

Possible: the timing of the reaction corresponds to the chronology of administration, the reaction corresponds to known reaction types of the investigational drug, the

patient's clinical status or other treatment modalities may also cause the reaction.

Possibly unrelated: The timing of the reaction does not correspond to the chronological order of administration, the reaction does not fit well with the known reaction type of the investigational drug, and the patient's clinical state or other treatment methods may also cause the reaction.

Irrelevant: The timing of the reaction does not correspond to the chronological order of administration, the reaction is consistent with the known reaction type of non-investigational drugs, the patient's clinical state or other treatment methods may also cause the reaction, the disease state improves or other treatment methods are discontinued, and the reaction disappears after repeated use of other treatment methods.

Unable to determine: the timing of the reaction is not clearly related to the chronological order of administration, the reaction is similar to the known reaction type of the investigational drug, and other drugs administered at the same time may also cause the same reaction.

Serious adverse event

Judgment of serious adverse event: death; life-threatening; leading to prolonged hospitalization; permanent or serious disability; leading to congenital malformation or defect.

End of Treatment (EOT) Visit

As specified in the protocol, post-treatment assessments will be done for all subjects approximately 1–3 days after the last dose of study drug. The timing of EOT assessments will be different for subjects who complete treatment and subjects who discontinue treatment early. Subjects that complete treatment will have those assessments done at the Week 8 visit and recorded on Week 8. Per protocol, subjects who discontinue treatment early will have those assessments done at an earlier visit and recorded on Early Termination.

To accurately summarize changes from baseline to end of treatment in safety endpoints, the EOT safety assessments for all subjects will be combined into a derived visit named “EOT”. If a subject discontinues treatment early but did not have data

recorded on Early Termination, then data recorded at the subject’s last visit during the treatment period will be used for the EOT visit. That visit will be included in summary tables of safety endpoints.

DATA ANALYSIS CONSIDERATIONS

Statistical analyses will be performed at the end of the trial after the last subject has completed the last visit, all data have been reported, monitored, cleaned, and the database has been locked. All data will be listed by arm, subject and visit/time point where appropriate. The summary tables will have columns corresponding to, or be stratified by arm.

Data will be summarized using descriptive statistics for continuous variables. Unless otherwise specified, descriptive statistics will include number of subjects (n), mean, standard deviation (SD), minimum, median and maximum. The minimum and maximum statistics will be presented to the same number of decimal places as the original data. The mean and median will be presented to 1 more decimal place than the original data, whereas the SD will be presented to two more decimal places than the original data.

In summary tables of categorical variables, counts and percentages will be used. The count [n] indicates the actual number of subjects with a particular value of a variable or event. Percentage will be obtained by: $\% = (n/M) \times 100$.

All dates in tables, figures and listings will be displayed in YYYY-MM-DD format.

Data Handling Rules

Baseline: The last non-missing observation (including unscheduled visits) prior to the first dose of study drug, unless otherwise specified. The last non-missing observation prior to the first dose of study drug, unless for randomized subjects who do not receive study drug then baseline is defined prior to date of randomization.

Dropouts will not be replaced during the study, but will be included in the data analysis to the extent that evaluable data are present. Mean imputations were used to find missing data during the treatment phase.

Analysis Sets

Three analysis sets will be used.

Screening Set

The Screening Set is defined as all subjects who give written informed consent and enter screening but are not randomized. Only demographic data and reasons for non-participation in the study will be summarized for this analysis set.

Efficacy Set

This is the All-Randomized Set, defined as all randomized subjects. Subjects will be evaluated by their randomized treatment arm (ITT analysis) unless otherwise specified. This set is referred to as the All-Randomized Set within this document.

Safety Set

The Safety Set is defined as all randomized subjects who take at least 1 dose of study drug. The Safety Set will be the primary set for safety summaries. Safety summaries will be provided based on the actual treatment received. Achieve will approve the list of subjects to be excluded from the Safety Set after database lock. Achieve must approve this list before the blind is broken and any analysis is performed.

Statistical methods

Continuous data will be expressed as arithmetic means ±standard deviation. Categorical variables will be expressed as frequencies. Comparative analysis of baseline characteristics between the groups will utilize the χ^2 test for categorical variables and *t* test for continuous variables.

Efficacy analysis

The efficacy analyses will be conducted using the All Randomized Set. Unless otherwise specified, all randomized subjects will be included in efficacy analyses. Subjects will be evaluated by their randomized treatment arm (ITT analysis).

The study will assess the treatment effects on primary and secondary outcome variables using a *t* test analysis, which will compare individual differences across treatment groups at different treatment phases.

Reference:

1. Moher D, Altman DG, Schulz KF, Simera I, Wager E. Spirit (standard protocol items: Recommendations for interventional trials). Toronto, Canada: John Wiley & Sons, Ltd; (2014). 56–67 p

2. Allen RE, Kirby KA. Nocturnal leg cramps. Am Fam Physician. 2012;86(4):350-355.
