# Supplementary material for: Vitamin K2 in Managing Nocturnal Leg Cramps: A Randomized Clinical Trial
Source: JAMA Intern Med. 2024 Oct 28;184(12):1443–7. doi: 10.1001/jamainternmed.2024.5726 (PMC11581596; doi:10.1001/jamainternmed.2024.5726)
Supplement: Supplement 3. — eTable 1. Overview of Outcomes Between the Vitamin K2 and the Placebo at Different Time Points eTable 2. Magnitude of Change From Baseline to Intervention Between Vitamin K2 and Placebo Group [file jamainternmed-e245726-s003.pdf]

Supplemental Online Content

Tan J, Zhu R, Li Y, et al. Vitamin K_2 in managing nocturnal leg cramps: a randomized clinical trial. *JAMA Intern Med*. Published online October 28, 2024. doi:10.1001/jamainternmed.2024.5726

eTable 1. Overview of Outcomes Between the Vitamin K_2 and the Placebo at Different Time Points
eTable 2. Magnitude of Change From Baseline to Intervention Between Vitamin K_2 and Placebo Group

This supplementary material has been provided by the authors to give readers additional information about their work.

© 2024 Tan J et al. *JAMA Internal Medicine*.

eTable1. Overview of outcomes between the vitamin K2 and the placebo at different time points					
End outcomes	Times	Mean (SD)		Mean (95%CI) for between arm change (Vitamin K2-Placebo)	*p*-value for between arm change
		Vitamin K2 (n=103)	Placebo (n=96)		
Frequency NLC/wk	Baseline	2.60(0.81)	2.71(0.80)	-0.12(-0.34,0.11)	
	Week 1	2.30(1.45)	3.39(2.17)	-1.08(-1.61, -0.56)	<0.0001
	Week 2	1.56(1.52)	3.98(2.12)	-2.42(-2.94, -1.90)	<0.0001
	Week 3	1.14(1.53)	3.42(2.06)	-2.28(-2.79, -1.77)	<0.0001
	Week 4	0.49(1.01)	3.57(2.36)	-3.08(-3.60, -2.57)	<0.0001
	Week 5	0.61(1.15)	3.52(2.33)	-2.91(-3.43, -2.38)	<0.0001
	Week 6	0.59(1.10)	3.63(2.29)	-3.04(-3.55, -2.54)	<0.0001
	Week 7	0.59(1.21)	3.85(2.18)	-3.26(-3.76, -2.76)	<0.0001
	Week 8	0.40(1.01)	3.71(2.10)	-3.31(-3.78, -2.84)	<0.0001
Duration of NLC, min	Baseline	1.15(0.89)	1.30(1.08)	-0.15(-0.42,0.13)	NA
	Week 1	0.66(0.68)	1.00(0.93)	-0.33(-0.56, -0.10)	NA
	Week 2	0.38(0.45)	1.03(1.15)	-0.65(-0.89, -0.40)	NA
	Week 3	0.31(0.60)	0.89(0.94)	-0.57(-0.80, -0.35)	NA
	Week 4	0.14(0.30)	0.92(1.00)	-0.78(-0.56, -0.10)	NA
	Week 5	0.14(0.32)	0.98(0.96)	-0.83(-1.03, -0.63)	NA
	Week 6	0.14(0.34)	0.93(0.90)	-0.79(-0.98, -0.60)	NA
	Week 7	0.13(0.32)	1.07(0.95)	-0.94(-1.14, -0.74)	NA
	Week 8	0.09(0.29)	1.02(0.99)	-0.92(-1.13, -0.71)	NA
Severity of NLC	Baseline	3.66(1.70)	3.32(1.19)	0.34(-0.07,0.75)	NA
	Week 1	2.74(2.03)	2.19(1.80)	0.55(0.01,1.09)	NA
	Week 2	1.71(1.77)	2.19(1.68)	-0.48(-0.96,0.01)	NA
	Week 3	1.32(1.71)	2.05(1.74)	-0.74(-1.22, -0.25)	NA
	Week 4	0.61(1.25)	1.99(1.72)	-1.38(-1.81, -0.96)	NA
	Week 5	0.70(1.38)	2.10(1.67)	-1.40(-1.83, -0.97)	NA
	Week 6	0.89(2.38)	1.92(1.80)	-1.03(-1.62, -0.44)	NA
	Week 7	0.57(1.30)	2.16(1.70)	-1.60(-2.02, -1.17)	NA
	Week 8	0.39(1.12)	2.05(1.71)	-1.66(-2.07, -1.25)	NA

© 2024 Tan J et al. *JAMA Internal Medicine*.

eTable2. Magnitude of change from baseline to intervention between vitamin K2 and placebo group

Outcomes	Mean (SD)						*P* value[b]
	Vitamin K2 (n=103)			Placebo (n=96)			
	Screening Phase	Intervention Phase	Change[a]	Screening Phase	Intervention Phase	Change[a]	
Frequency of NLCs/wk	2.60(0.81)	0.96(1.41)	-1.64(1.57)	2.71(0.80)	3.63(2.20)	0.92(2.04)	<0.0001
Duration of NLCs, min	1.15(0.89)	0.25(0.47)	-0.90(0.88)	1.30(1.08)	0.98(0.98)	-0.32(0.78)	NA
Severity of NLCs	3.66(1.70)	1.12(1.82)	-2.55(2.12)	3.32(1.19)	2.08(1.72)	-1.24(1.16)	NA

Abbreviations: NLCs, nocturnal leg cramps; Wk, week; SD, Standard Deviation; NA,

[a] Change indicates the difference in the mean outcome of NLCs between the screening and intervention phase.

[b] *P* value indicates the mean change of frequency from baseline to intervention between Vitamin K2 vs. placebo group.

© 2024 Tan J et al. *JAMA Internal Medicine*.
