# Supplementary material for: Vitamin K2 in Managing Nocturnal Leg Cramps: A Randomized Clinical Trial
Source: JAMA Intern Med. 2024 Oct 28;184(12):1443–7. doi: 10.1001/jamainternmed.2024.5726 (PMC11581596; doi:10.1001/jamainternmed.2024.5726)
Supplement: Supplement 4. — Data Sharing Statement [file jamainternmed-e245726-s004.pdf]

Data Sharing Statement

Tan. Vitamin K_2 in Managing Nocturnal Leg Cramps. *JAMA Intern Med*. Published December 02, 2024. doi:10.1001/jamainternmed.2024.5726

Data
Additional Information: clinicaltrials.gov Identifier: NCT05547750
Data available: Yes
Data types: Deidentified participant data
How to access data: 79468064@qq.com
When available: With publication

Supporting Documents
Document types: Statistical/analytic code
How to access documents: 79468064@qq.com
When available: With publication

Additional Information
Who can access the data: anyone requesting the data, researchers whose proposed use of the data has been approved
Types of analyses: for study purpose
Mechanisms of data availability: after approval of a proposal, or with a signed data access agreement
Any additional restrictions: None
